# Downregulation of tumor‐derived exosomal miR-34c induces cancer‐associated fibroblast activation to promote cholangiocarcinoma progress

**DOI:** 10.1186/s12935-020-01726-6

**Published:** 2021-07-14

**Authors:** Xinglei Qin, Min Lu, Gang Li, Yajun Zhou, Zhaoyang Liu

**Affiliations:** 1 Department of Hepatobiliary Surgery, Henan Provincial People’s Hospital, Zhengzhou University People’s Hospital, Henan University People’s Hospital, No.7 Weiwu Road, Zhengzhou, Henan 450003 China; 2 Department of Cardiology, Henan Provincial People’s Hospital, Zhengzhou University People’s Hospital, Henan University People’s Hospital, Zhengzhou, Henan 450003 China

**Keywords:** Cholangiocarcinoma, Fibroblasts, Exosomes, miR-34c, WNT1

## Abstract

**Background:**

This study aimed to investigate the exact regulatory mechanisms of exosomal miR-34c in mediating communication between cholangiocarcinoma cells and fibroblasts.

**Methods:**

Exosomes were isolated from HuCCT-1 and HIBEC cells using differential ultracentrifugation and identified by transmission electron microscopy (TEM) and nanoparticle tracking analysis (NTA) method. Real-time quantitative PCR (qRT-PCR) and western blotting analyses were performed to assess the levels of pro-inflammatory factors, and fibroblast-related proteins and Wnt-linked signaling pathway proteins, respectively. Exosome-tracking was performed with confocal microscopy. The 3-[4,5-dimethylthiazol-2-yl]-2,5-diphenyl tetrazolium bromide (MTT) and Transwell assays were used to measure cell proliferation and migration, respectively. Further, the oncogenicity of cholangiocarcinoma cells was analyzed in nude mice transplanted tumor model.

**Results:**

The analysis suggested that the expression of miR-34c was decreased in exosomes from HuCCT-1 cells. Moreover, miR-34c in exosomes mediated fibroblast activation by directly targeting WNT1. Additionally, cancer-associated fibroblasts (CAFs) activated by downregulation of exosomal miR-34c promoted cholangiocarcinoma progression.

**Conclusions:**

Thus, miR-34c in exosomes was found to be a key player in regulating intercellular communication between tumor cells and fibroblasts.

## Background

Cholangiocarcinoma (CCA) is a malignancy arising from the epithelial cells of the biliary tree, comprising 3% of all the gastrointestinal malignancies [1, 2]. It is classified into intrahepatic cholangiocarcinoma (iCCA) and extrahepatic cholangiocarcinoma (ECC) subtypes based on the anatomical location. ECC includes perihilar cholangiocarcinoma (pCCA) and distal cholangiocarcinoma (dCCA). The development of CCA is caused by multiple factors, such as hepatitis B and C infection, underlying primary sclerosing cholangitis, biliary stone disease, congenital biliary cysts, and liver fluke infection [3–5]. CCA is characterized by late onset, significant invasiveness, distant metastasis, and poor prognosis [6–8]. Several studies have shown that components of tumor microenvironment (TME) play an important role in tumor metastasis [9–13]. Tumor cells influence their adjacent microenvironment at an earlier stage in malignant progression through cell–cell interaction and paracrine mechanisms.

The major cell types involved in the tumor microenvironment are—fibroblasts, endothelial cells, neuroendocrine cells, adipose cells, and immune-inflammatory cells—which affect the tumor characteristics, such as growth, dormancy, invasion, and metastatic growth [14]. In particular, CAFs, as an activated subpopulation of fibroblasts, are the most common risk factor for tumor progression and metastasis [15, 16]. Moreover, CAFs express α-smooth muscle actin (α-SMA) and fibroblast-activated protein (FAP) [16]. Studies suggest that pro-inflammatory proteins secreted by CAFs promote an antitumor immune response or support tumor pathogenesis by regulating the inflammatory microenvironment [17, 18]. However, the mechanism underlying the activation of fibroblasts in cholangiocarcinoma remains unknown.

A previous study has indicated that exosomes (diameter, 30–150 nm) are nanometer-sized vesicles with a phospholipid bilayer structure, and are secreted from the endosomal membrane compartment of diverse cell types [19]. Exosomes are composed mainly of nucleic acids, proteins, and lipids. These functional biomolecules can be delivered to recipient cells, and participate in a variety of physiological and pathological processes, including immunomodulation, cancer progression, and epigenetic reprogramming [20–22].

MicroRNAs (miRNAs) are endogenous small non-coding RNAs that function as key post-transcriptional gene expression regulators by binding to their target mRNAs [23, 24]. Moreover, regulation of miRNAs in CAFs has been studied during carcinogenesis [25–27]. Several reports have indicated that exosome-containing miRNAs are involved in many cancer-related events, functioning either as oncogenes or tumor suppressors [28–31]. Tumor cell-derived exosomes mediated miRNA transfer is an important mechanism in tumor microenvironment by uptake and internalized-exosomes. Yang et al. [32] have shown that HCC-derived exosomes transfer their contents (such as miRNAs) into recipient cells to mediate transmission of fungt1onal transgenes mediate transmission involved HCC growth and progress. Kitdumrongthum et al. [33] found that miR-34c present a remarkably low expression in exosomes isolated from CCA cell lines. In addition, under-expression of miR-34c was observed in cholangiocarcinoma-associated fibroblasts compared with fibroblasts from non- cancer tissues [34]. The impact of exosomal miRNAs underlying effect via CCA-associated fibroblasts transmit information to cholangiocarcinoma has not so far been reported.

This study aimed to explore the exosomal miRNA-mediated crosstalk between CAFs and cholangiocarcinoma cells. First, we derived exosomes from normal and cancerous cells. Further, we performed experiments, including exosomal miRNA arrays, to verify the effect of miRNAs delivered via tumor-derived exosomes in regulating activation of fibroblast. Moreover, we validated that miR-34c was directly transferred through cancer-cell derived exosomes, from CAFs to cholangiocarcinoma cells, and identified the molecular mechanisms by which exosomal miR-34c modulates migration and invasion in tumor cells.

## Materials and methods

### Cell culture and transfection

Human cholangiocarcinoma cell lines (HuCCT-1, CCA; QBC939), normal human skin fibroblasts (CCC-HSF-1), and normal human cholangiocyte cell line (HIBEC) were purchased from the BeNa culture collection (BNCC, Beijing, China). The QBC939, CCC-HSF-1 and HIBEC cells were grown in Dulbecco’s Modified Eagle’s medium (DMEM; Invitrogen Corporation, CA, USA), while HuCCT-1 cells were cultured in RPMI-1640; all cells were supplemented with 10% fetal bovine serum (FBS; Gibco; Thermo Fisher Scientific, Inc.) and 1% antibiotics (100 U/mL penicillin, and 100 μg/mL streptomycin) (Gibco; Thermo Fisher Scientific, Inc.) and incubated at 37 °C and 5% CO_2_ − 95% air in a humidified chamber. The CCC-HSF-1 cells were treated with exosomes (10 µg/mL) derived from HIBEC and HuCCT-1 cells in the presence or absence of miR-34c mimic for 24 h.

The hsa-miRNA mimics were commercially available (Ruibo Biotechnology Co., Guangzhou, China). The product number miR10000686-1-5 was used as a miR-34c mimic. The scrambled miRNA mimic was used as the negative control miRNA mimic. The hsa-miRNA inhibitor (miR-34c inhibitor and a scrambled miRNA inhibitor, and siRNAs (si NC, siRNA targeting Wnt,) were obtained from Ruibo Biotechnology. The HuCCT-1 and CCC-HSF-1 cells were transfected with 50 nM hsa-miR-34c mimics and negative control miRNA mimics using Lipofectamine RNAi Max (Invitrogen, Carlsbad, CA) according to the manufacturer’s protocol. Additionally, CCC-HSF-1 cells were transfected with 50 nM miR-34c inhibitor and inhibitor NC, or si-Wnt and si-NC using the Lipofectamine RNAi Max. After transfection for 48 h, the transfection efficiency of miRNA mimics, inhibitors, or siRNAs was confirmed at the RNA level using real-time PCR.

### Luciferase reporter assay

The 3′-UTR of human *WNT1*, including conserved binding sites for miR-34c-5p, was PCR amplified from human cDNA and integrated into a pGL3 vector. A mutant 3′-UTR fragment of *WNT1*, in which the mutation was in the conserved binding sites for miR-34c-5p (GUGACGG), was also generated. These sequences, including the wild-type 3′-UTR regions (3′-UTR-WT) or mutant 3′-UTR regions (3′-UTR-Mut) of *WNT1*, were inserted into the p-Luc-UTR vector. Further, the constructed vectors were cotransfected into CCC-HSF-1 cells with NC or miR-34c-5p mimics using Dharmafect Duo transfection reagent (Thermo Fisher Scientific). After 48 h, the cells were collected, and luciferase activities were determined using a Dual-Luciferase reporter assay system (Promega, Madison, WI) according to the manufacturer’s instructions.

### Exosome isolation

Differential centrifugation was performed to obtain and purify exosomes from the culture supernatants. Briefly, cell lines (NCC-Exo, HIBEC; CCA-Exo, HuCCT-1; NCC-Exo, HuCCT-1-mimic NC; and miR-34 cM-Exo, HuCCT-1-miR-34c mimics) were cultured in 10-cm plates containing fresh complete medium supplemented with 10% exosome-depleted FBS, which was centrifuged at 12,000×*g* overnight. The culture media were collected after cells reached approximately 70% confluence. Further, cellular debris in the supernatants was removed by centrifugation at 1500×*g* for 10 min, followed by re-centrifugation at 10,000×*g* for 30 min at 4 °C. The supernatants were obtained and filtered using a 0.22 μm filter (Millipore, USA), and then ultracentrifuged at 110,000×*g* for 90 min (Optima MAX-XP, Beckman Coulter, USA). The pellets were washed with PBS and centrifuged at 110,000×*g* for 70 min. The Pierce BCA Protein Assay Kit was used to measure the total protein concentration in exosomes. The isolated exosomes were suspended in PBS, and stored at − 80 °C until use.

### Exosome characterization

TEM (Philips CM120 BioTwin, FEI Company, USA) was used to observe morphology of the isolated exosomes. Briefly, the isolated exosomes were fixed in 2% paraformaldehyde and spotted onto a copper grid (EM Resolutions, Saffron Walden, UK), which was dried for 15 min at room temperature. Further, the samples were stained with 2% uranyl acetate for 10 min and examined by TEM at 80 keV. The nanoparticle tracking analysis (NTA) method was used to characterize the size distribution of the isolated exosomes using NanoSight NS300 (Malvern Instruments Ltd, UK) following the manufacturer’s instructions.

### Exosomes tracking

For exosome-tracking experiments, exosomes derived from HuCCT-1 cells were labeled with the lipophilic dye DiO green (Beyotime Biotechnology, China). After incubating recipient cells (CCC-HSF-1) pretreated with Dil (red, Beyotime) for 24 h, presence of exosomes from HuCCT-1 cells was observed in recipient cells using confocal laser scanning microscopy (TCS SP5; Leica Microsystems, Wetzlar, Germany).

### Confocal microscopy

DiO-labeled exosomes were added to HuCCT-1 cells transfected with FAM-miR-34c-M (miR-34c mimics) for 24 h at 15 μg/mL concentration in a 6-well plate. Cells were washed with PBS and fixed with ice-cold acetone-methanol (1:1) for 5 min, and then washed with PBS. Cells were imaged using the Leica TCS SP5 confocal microscope.

### In vivo experiments

Male BALB/c nude mice (6–7-week old) were purchased from the Shanghai Experimental Animal Center (Shanghai, China), and housed in a pathogen-free environment. All experimental protocols involving animals were approved by the Institutional Animal Care and Use Committee of Henan Provincial People’s Hospital, China (Ethical approval Number: JN.No20181211c0510105 [302]). A total of 32 nude mice were randomly divided into four groups (n = 8, each), and subcutaneously implanted with QBC939 (5.0 × 10^6^) cells suspended in 0.1 mL saline. First, CCC-HSF-1 cells were pretreated with exosomes from HIBEC and HuCCT-1 cells with or without miR-34c mimics and NC mimics. Further, QBC939 cells were incubated with the supernatant of CCC-HSF-1. The treatment groups were as follows: HIBEC-Exo (NCC-Exo), HuCCT-1-Exo (CCA-Exo), HuCCT-1-mimics NC-Exo (NC-Exo), and HuCCT-1-miR-34c mimic-Exo (miR-34 cM-Exo). Tumor growth was monitored every 7 days using an electronic caliper until the animals were euthanized and tumors were excised on the 42th day of the experiment.

$${\text{Tumor}} {\text{volume}}\, = \,\left( {{\text{shortest}} {\text{diameter}}} \right)^{ 2} \, \times \,\left( {{\text{longest}} {\text{diameter}}} \right)\, \times \,0. 5.$$

### RNA extraction and quantitative real-time PCR (qRT-PCR)

TRIzol reagent (Invitrogen, USA) and miRNeasy RNA isolation kit (QIAGEN, Hilden, Germany) were used for isolation of total RNA from cells and secreted exosomes, according to the manufacturer’s instructions. Reverse transcription was performed using a cDNA Reverse Transcription Kit (TaKaRa Bio, Japan) and miScript II RT kit (QIAGEN, Hilden, Germany) for mRNA and miRNA, respectively. Next, qRT-PCR analysis was performed with the SYBR Green PCR Master Mix (Invitrogen, USA) for mRNA, and miScript SYBR Green PCR kit (QIAGEN, Hilden, Germany) with SYBR Green Realtime PCR Master Mix (TOYOBO, Japan) for miRNA. The qRT-PCR amplification was performed at 95 °C for 60 s, followed by 40 cycles at 95 °C for 10 s and 60 °C for 30 s; and the relative mRNA and miRNA expression was evaluated using the 2^−ΔΔCT^ calculation method and normalized with β-actin and *18* *s rRNA*, respectively. Primers are shown in Table 1.

**Table 1:** PCR primer sequences

IL-1B	Forward Sequence CCACAGACCTTCCAGGAGAATG
	Reverse Sequence GTGCAGTTCAGTGATCGTACAGG
IL-6	Forward Sequence AGACAGCCACTCACCTCTTCAG
	Reverse Sequence TTCTGCCAGTGCCTCTTTGCTG
IL-8	Forward Sequence GAGAGTGATTGAGAGTGGACCAC
	Reverse Sequence CACAACCCTCTGCACCCAGTTT
miR-34c	Forward Sequence GGCAGTGTAGTTAGCTG
	Reverse Sequence GAACATGTCTGCGTATCTC
WNT1	Forward Sequence CTCTTCGGCAAGATCGTCAACC
	Reverse Sequence CGATGGAACCTTCTGAGCAGGA
β-actin	Forward Sequence CACCATTGGCAATGAGCGGTTC
	Reverse Sequence AGGTCTTTGCGGATGTCCACGT
18 s rRNA	Forward Sequence ACCCGTTGAACCCCATTCGTGA
	Reverse Sequence GCCTCACTAAACCATCCAATCGG

### Enzyme-linked immunosorbent assay (ELISA)

The levels of cytokines in CCC-HSF-1 supernatant including interleukin (IL)-1β, interleukin (IL)-6 and interleukin (IL)-8 were measured with their commercial ELISA kits (R&D Systems, USA) following the manufacturer’s instruction.

### Western blotting

Total protein lysates were extracted from CCC-HSF-1 cell lines using lysis buffer (Beyotime, Shanghai, China). Bicinchoninic acid (BCA) Assay Kit (Thermo Fisher Scientific, USA) was used to test the protein concentrations. Equal amounts of protein or exosome samples were separated using 10% sodium dodecyl sulfate polyacrylamide gel electrophoresis (SDS-PAGE) and electro-transferred onto polyvinylidene fluoride (PVDF) membranes (Millipore Corp., Billerica, MA, USA). The nonspecific protein binding in the membranes was blocked by incubation with 5% nonfat milk for 1 h at room temperature. Further, the membranes were incubated with primary antibodies overnight at 4 °C, and washed three times with TBST. The membranes were then incubated with the horseradish peroxidase-conjugated secondary antibody (1:3000; Beyotime Biotechnology, China) at room temperature for 1 h. The enhanced chemiluminescence reaction (Thermo Scientific) was used to detect the protein bands. The primary antibodies used were as follows: anti-α-SMA (Abcam, Cambridge, MA, USA, 1:1000), anti-FAP (Abcam, 1:500), anti-WNT1 (Abcam, 1:1000), and anti-phospho-β-catenin (p-β-catenin, 1:500 dilution) and β-catenin (1:1000; CST Biological Reagents Co., Ltd., Shanghai, China). For assessing the protein markers of exosomes, antibodies against CD9 (1: 1000) and CD81 (1:1000) were purchased from Abcam (Cambridge, MA, USA), and CD63 (1:1000) from Santa Cruz Biotechnology (Santa Cruz, CA, USA).

### Cell proliferation assay

The proliferation of QBC939 cells was assessed using an MTT assay kit (Beyotime, China) following the manufacturer’s protocol. Briefly, the treated cells were seeded into 96-well plates at a density of 2 × 10^3^ cells/well. After 12, 24, 48, and 72 h, 20 μL MTT (5 mg/mL, Sigma-Aldrich, MO, USA) was added into each well and incubated without light for 4 h. The culture medium was then replaced with 150 μL dimethyl sulfoxide (DMSO) for 10 min to solubilize the crystals. Cell proliferation was assessed by measuring the optical density (OD) using a microplate reader (iMark 680; Bio-Rad Laboratories, Inc., Hercules, CA, USA) at a wavelength of 490 nm. Experiments were performed in triplicates, and all measurements were repeated a minimum of three times.

### Cells migration assay

QBC939 (2 × 10^4^) cells were suspended in serum-free DMEM, and added to the upper chamber of 24-well Transwell plates (Corning). Whereas, complete-serum medium containing 600 μL DMEM with 10% FBS was added to the bottom chamber. After 24 h, cells adhering to the bottom of the chamber were fixed with methanol and stained using 0.5% crystal violet for 15 min (Sigma). The stained cells were photographed under a microscope (Nikon E100; Nikon Corp, Tokyo, Japan), and the cell number was counted from to 5–8 random fields in each well.

### Statistical analysis

SPSS 19.0 software (IBM, Armonk, NY, USA) was used for performing the statistical analyses. The significance between two groups was analyzed using the unpaired Student’s *t* test. The differences between multiple groups were analyzed by one-way ANOVA. P < 0.05 was considered significant. All results are presented as mean ± standard deviation (SD) of at least three independent experiments.

## Results

### Tumor-derived exosomes regulate activation of CAFs

**Fig. 1:**
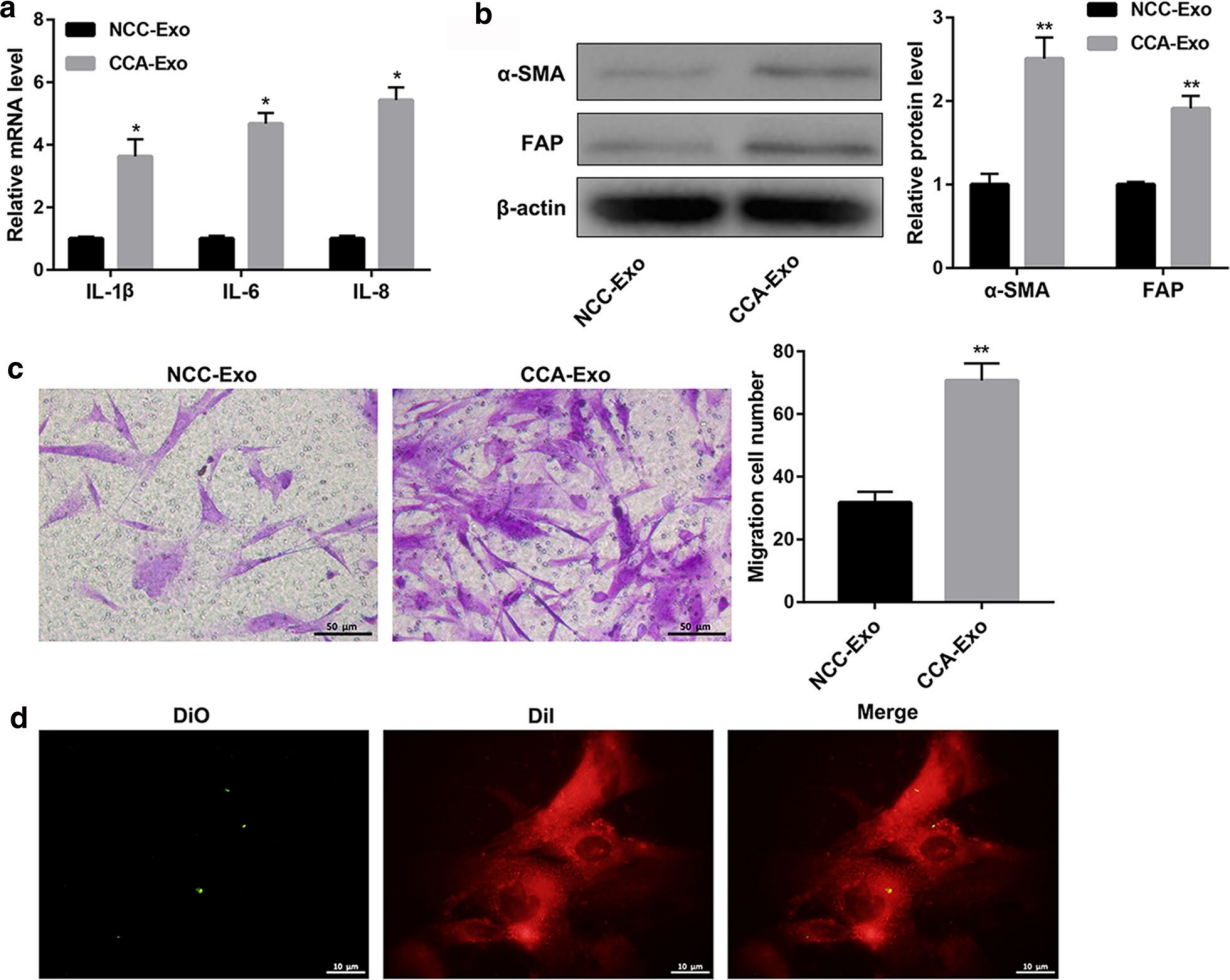
Exosomes secreted from cancer cells regulate activation of fibroblasts. **a** qRT-PCR analysis of expression of pro-inflammatory genes—IL-1β, IL-6, and IL-8—in CCC-HSF-1 cells treated with exosomes from HuCCT-1 or HIBEC cells. **b** Western blotting analysis of fibroblast activation proteins—α-SMA and FAP. **c** Migration assay for CCC-HSF-1 cells treated with equal quantities of exosomes derived from tumor or normal cells; Scale bar, 50 μm. **d** Confocal imaging of Dio-labeled (green) exosomes incubated with Dil-labeled (red) fibroblasts; Scale bar, 10 µm. Error bars, SD. *P < 0.05, **P < 0.01

First, we performed differential ultracentrifugation to isolate exosomes from HuCCT-1 and HIBEC cells. The enriched exosomes pellets were visualized using TEM, which demonstrated a uniformly round-shaped morphology with a size distribution of 40–150 nm (Additional file 1). Based on NTA, isolated exosomes showed a modal size of 110 ± 1.5 nm (Additional file 2: Fig. 2a). Additionally, western blotting analysis confirmed presence of CD9, CD63, and CD81 in exosomes isolated from HuCCT-1 and HIBEC cells (Additional file 2: Fig. 2b). These results confirmed that the vesicles derived from these cell lines were exosomes. Exosomes released from tumor cells can induce changes in the tumor microenvironment, which raises the question whether exosomes can promote activation of fibroblasts. Thus, we assessed the effect of exosomes derived from HuCCT-1 cells on activation of CCC-HSF-1 cells. The fibroblasts treated with exosomes showed a significant increase in the mRNA and protein levels of IL-1β, IL-6, and IL-8 than that in controls, as demonstrated by qRT-PCR and ELISA analysis (Fig. 1a and Additional file 3: Fig. 3a). Moreover, western blotting analysis indicated significantly increased expression of α-SMA and FAP in the exosome-pretreated group (CCA-Exo) than that in the control group (NCC-Exo) (Fig. 1b). Furthermore, as observed in Transwell assay, increased number of fibroblasts migrated in the CCA-Ex group than that in the control group (Fig. 1c). Further, exosome tracking assay showed presence of Dio spots (green) in the recipient Dil-labeled fibroblasts (red), confirming that labeled-exosomes released from tumor cells had entered the fibroblasts (Fig. 1d). Taken together, these results suggest that exosomes derived from HuCCT-1 cells contribute to activation of fibroblasts.

### miR-34c mediates fibroblast activation in exosomes

**Fig. 2:**
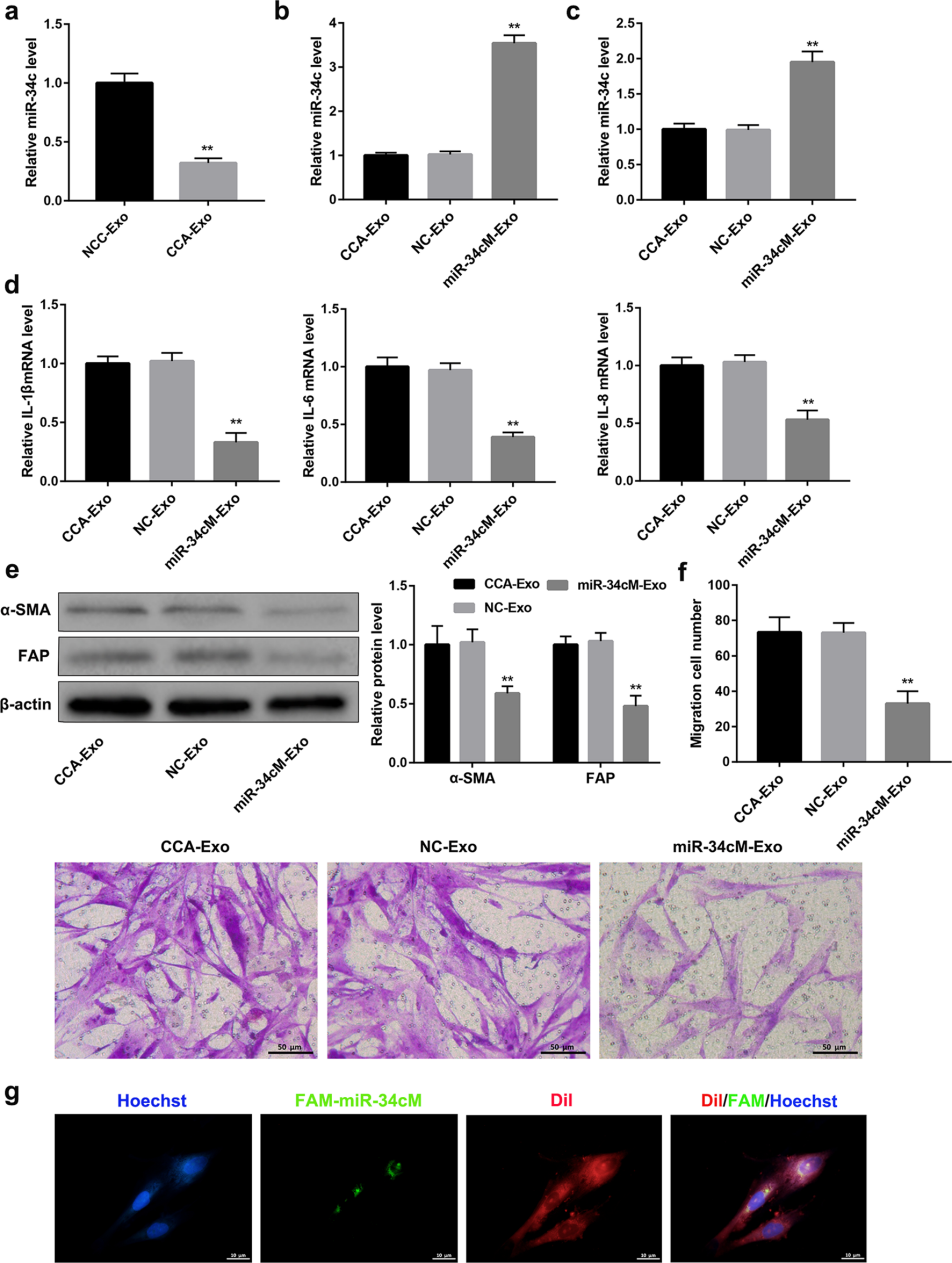
Exosomal miR-34c affects activation of fibroblasts. **a** qRT-PCR analysis of levels of miR-34c in exosomes derived from HIBEC and HuCCT-1 cells. **b** qRT-PCR analysis of levels of miR-34c in exosomes derived from HuCCT-1 cells treated with or without miR-34c mimics. **c-g** CCC-HSF-1 cells are incubated with exosomes for 48 h. qRT-PCR analysis of levels of **c** miR-34c, and **d** IL-1β, IL-6, and IL-8. **e** Western blotting analysis of α-SMA and FAP proteins. **f** Representative images and quantification of cell migration in CCC-HSF-1 cells; Scale bar, 50 μm. **g** Representative confocal images for immunofluorescence staining indicating delivery of FAM-labeled miR-34c (green) to Dil-labeled CCC-HSF-1 cells (red). Co-localized areas, representing delivery of exosomes, appear in yellow; Scale bar, 10 μm. **P < 0.01 *vs.* NC-Exo groups

miRNAs enclosed in exosomes are recognized as important mode of communication between the cells [35]. Thus, we explored whether the secreted miRNAs participate in activation of fibroblasts by tumor-derived exosomes. qRT-PCR was used to analyze the expression of specific miRNAs involved in exosomes. The results showed that expression of miR-34c to be significantly increased in the HuCCT-1-exosomes group than that in the control group (Fig. 2a). Additionally, levels of miR-34c were significantly increased in exosomes from HuCCT-1 cells transfected with miR-34c mimic (miR-34 cM-Exo) than that in the mimic-NC (NC-Exo) transfection group and blank group (CCA-Exo), indicating successful miR-34c over-expression in HuCCT-1 cells (Fig. 2b), and incubation of CCC-HSF-1 cells with these exosomes resulted in increased expression of miR-34c than that in the control group (Fig. 2c). Moreover, miR-34c overexpression in CCC-HSF-1 cells significantly suppressed the expression of IL-1β, IL-6, and IL-8 than that in control group (Fig. 2d and Additional file 3: Fig. 3b). In addition, the western blotting results suggested that increased expression of miR-34c significantly reduced the levels of α-SMA and FAP than that in the control group (Fig. 2e). Furthermore, migration assay showed reduction in number of migratory cells in the miR-34c treatment group than in the control group (Fig. 2f), reflecting that miR-34c mimic could also suppress the migratory potential of the fibroblasts. Further, immunofluorescence co-localization analysis confirmed that FAM-labeled miR-34c (green) was delivered by Dil-labeled fibroblasts (red), suggesting that miR-34c enclosed in exosomes plays an important role in cell–cell communication (Fig. 2g). These findings reveal that tumor-derived exosomal miR-34c inhibits fibroblast activation.

### Exosomal miR-34c targets WNT1 in CAFs

**Fig. 3:**
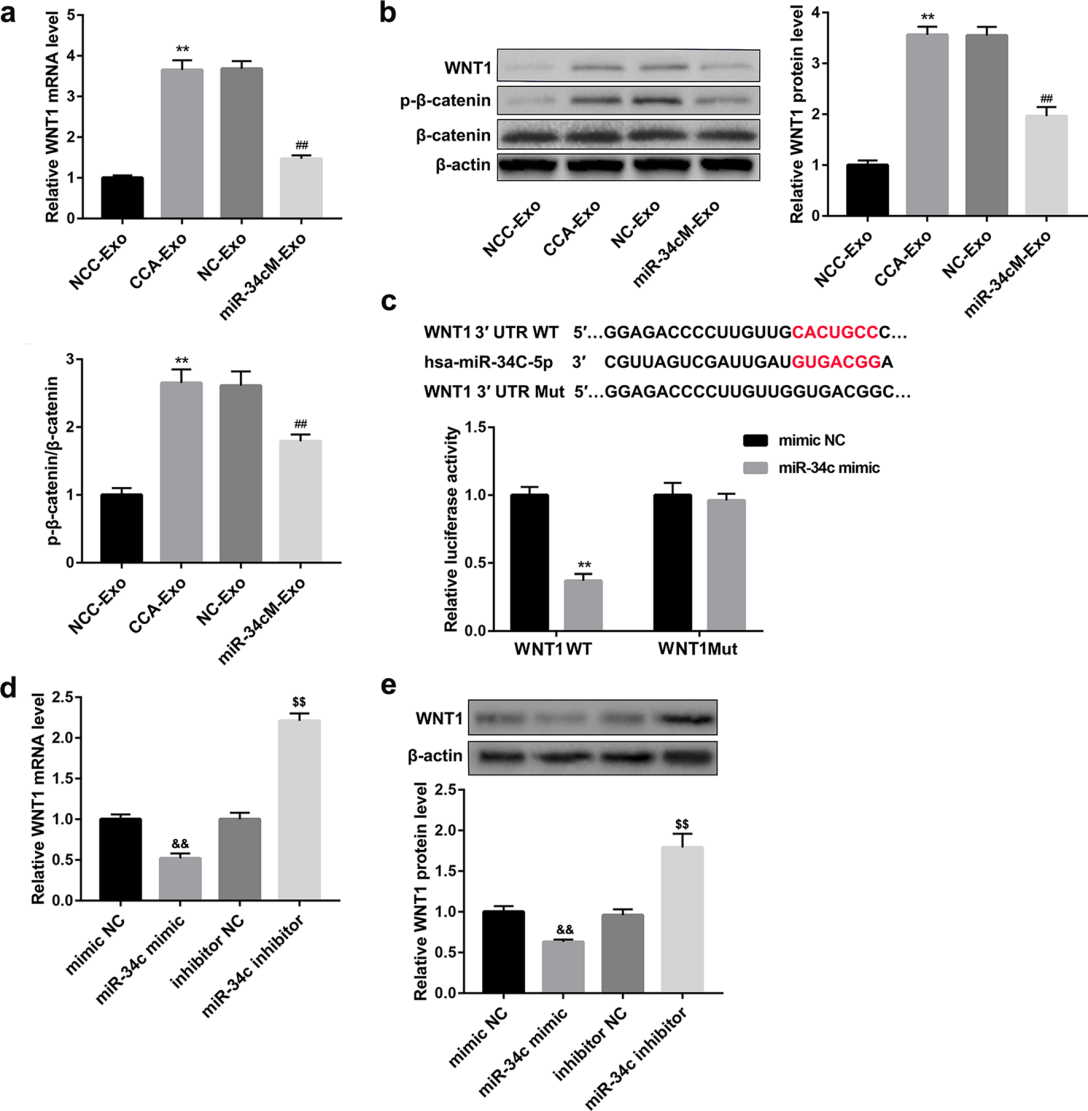
Exosomal miR-34c inhibits fibroblast activation by targeting WNT1 (**a, b**) qRT-PCR analysis of WNT1 and western blotting analysis of WNT1, β-catenin and p-β-catenin in CCC-HSF-1 cells treated with or without miR-34 mimics and control. **c** Relative luciferase activity between wild-type and mutant-type binding site of miR-34c and WNT1. (**d, e**) Transcript and protein levels of WNT1 assessed in CCC-HSF-1 cells in the presence or absence of miR-34c mimics or inhibitor. **P < 0.01 *vs.* NCC-Exo groups;##P < 0.01 *vs.* NC-Exo groups; ^&&^P < 0.01 *vs.* mimic NC groups; ^$$^P < 0.01 *vs.* inhibitor NC groups

Next, to identify the targets of exosomal miR-34c in fibroblasts, bioinformatics tools were used to predict a set of common target genes. The analysis indicated WNT1 to be a direct target of miR-34c, and contributes to activation of fibroblast. Additionally, the analysis identified a miR-34c binding site in the WNT1 sequence. Further, we observed that expression of WNT1 was upregulated in CCC-HSF-1 cells treated with exosomes from cancer cells, and downregulated in miR-34c-loaded exosomes at both the mRNA and protein levels (Fig. 3a, b). The p-β-catenin levels were concordant with that of WNT1 (Fig. 3b). Moreover, luciferase reporter assay indicated significant decrease in luciferase activity after co-transfection of the miR-34c mimics and luciferase reporter gene with the wild-type binding site vector (Fig. 3c). However, the mutated binding site vector group showed no difference in luciferase activity between treatment with miR-34c mimics and NC-mimics. Furthermore, overexpression of miR-34c with mimics suppressed the expression of WNT1, but inhibition of miR-34c elevated expression of WNT1, as confirmed by qRT-PCR and western blotting analysis (Fig. 3d, e). Thus, these results suggest WNT1 as a direct target of miR-34c in fibroblasts.

### miR-34c suppresses activation of CAFs by targeting Wnt signaling pathway

**Fig. 4:**
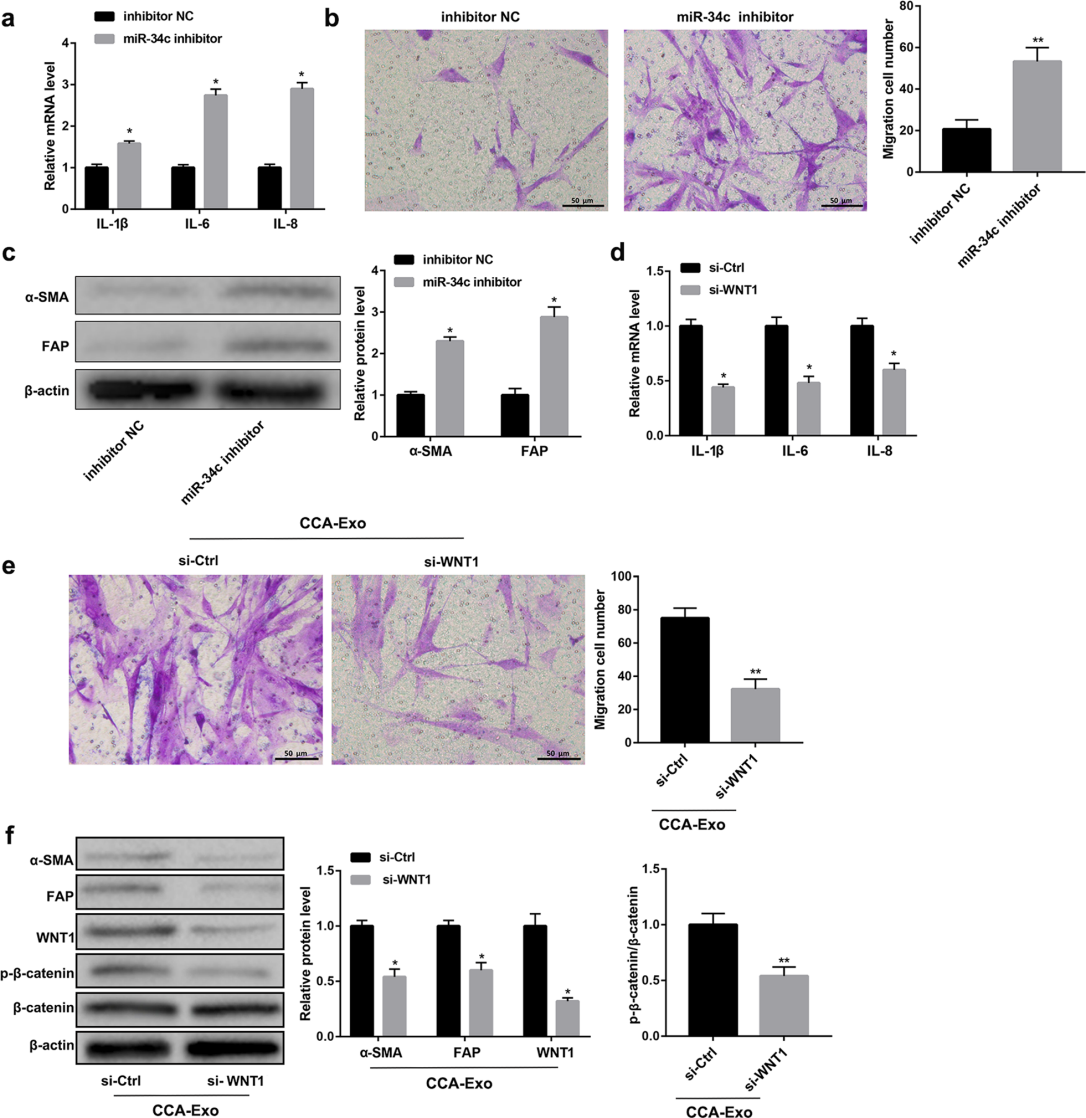
Inhibition of miR-34c restores Wnt signaling pathway and activates fibroblasts. **a-c** qRT-PCR, western blotting, and migration assay analyses to assess expression of pro-inflammatory genes, protein levels of α-SMA and FAP, and cell migration, respectively, in CCC-HSF-1 cells treated with or without miR-34c inhibitor. **d-f** qRT-PCR, western blotting, and cell migration assay analyses to detect expression of pro-inflammatory genes, levels of fibrinogen and Wnt pathway-related proteins, and cell migration, respectively, in CCC-HSF-1 cells treated with or without si-WNT1 and CCA-Exo; Scale bar, 50 µm. ^*^P < 0.05, ^**^P < 0.01 *vs.* inhibitor NC or si-Ctrl groups

**Fig. 5:**
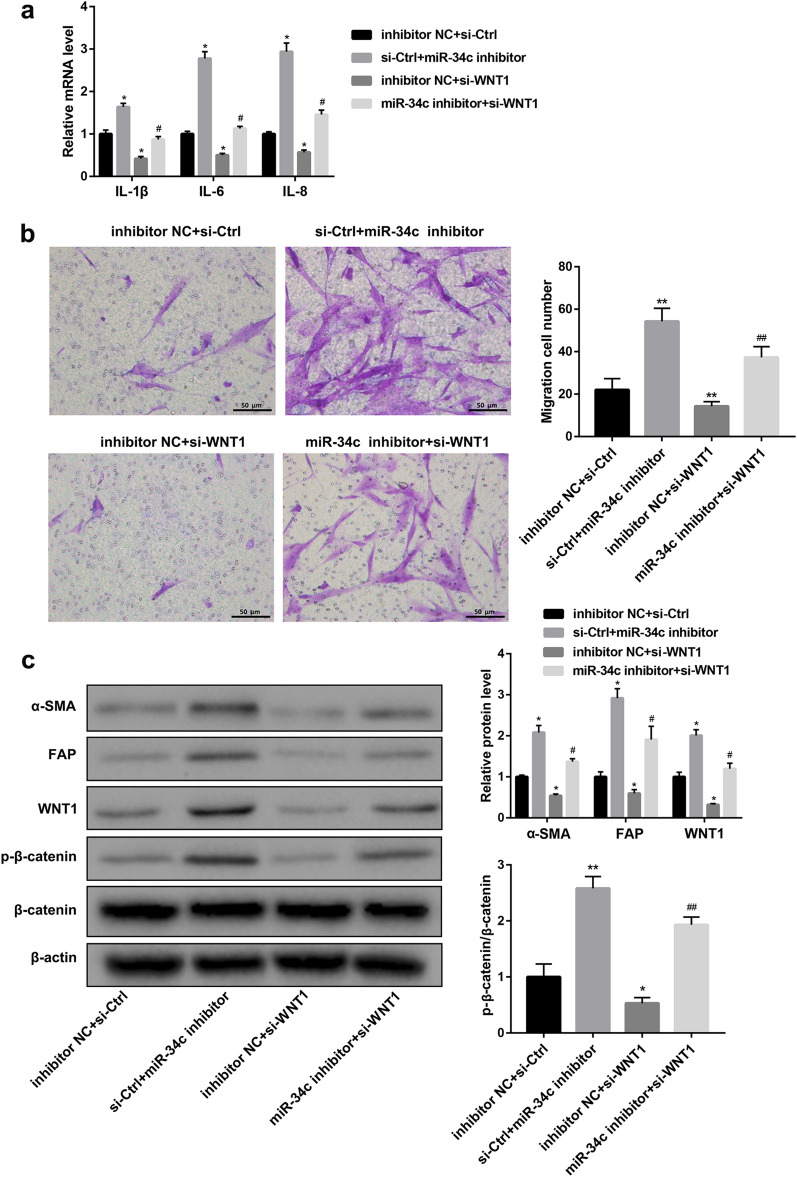
WNT1 suppression reverses the effect of miR-34c inhibition. **a** qRT-PCR analysis of expression of IL-1β, IL-6, and IL-8 in CCC-HSF-1 cells transfected with miR-34c inhibitor and/or WNT1 siRNA. **b** Migration assay analysis of CCC-HSF-1 cells transfected with miR-34c inhibitor and/or WNT1 siRNA or control. **c** Western blot analysis of fibrinogen and Wnt pathway-related proteins; Scale bar, 50 µm. *P < 0.05, **P < 0.01 *vs.* inhibitor NC + si-Ctrl groups;^#^P < 0.05, ^##^P < 0.01 *vs.* inhibitor NC + si-WNT1 groups

Next, we checked the effect of miR-34c–WNT1 signaling axis on the activation of fibroblasts. As shown in Fig. 4a–c and Additional file 3: Fig. 3c, inhibition of miR-34c promoted pro-inflammatory expression, cell migration, and activation of fibroblasts-related proteins in CCC-HSF-1 cells than that in control cells, but contrary results were obtained when CCC-HSF-1 cells were transfected with si-WNT1 and treated with CCA-Exo (Fig. 4d–f and Additional file 4). Moreover, siRNA-interference of WNT1 significantly inhibited the levels of Wnt signaling pathway proteins (Wnt and p-β-catenin) (Fig. 4f). However, the co-expression of miR-34c and WNT1 reversed the above results (Fig. 5 and Additional file 3: Fig. 3d). Taken together, these data suggest that the downregulation of miR-34c activates Wnt signaling pathway in fibroblasts.

### Activated CAFs promote cholangiocarcinoma progression

**Fig. 6:**
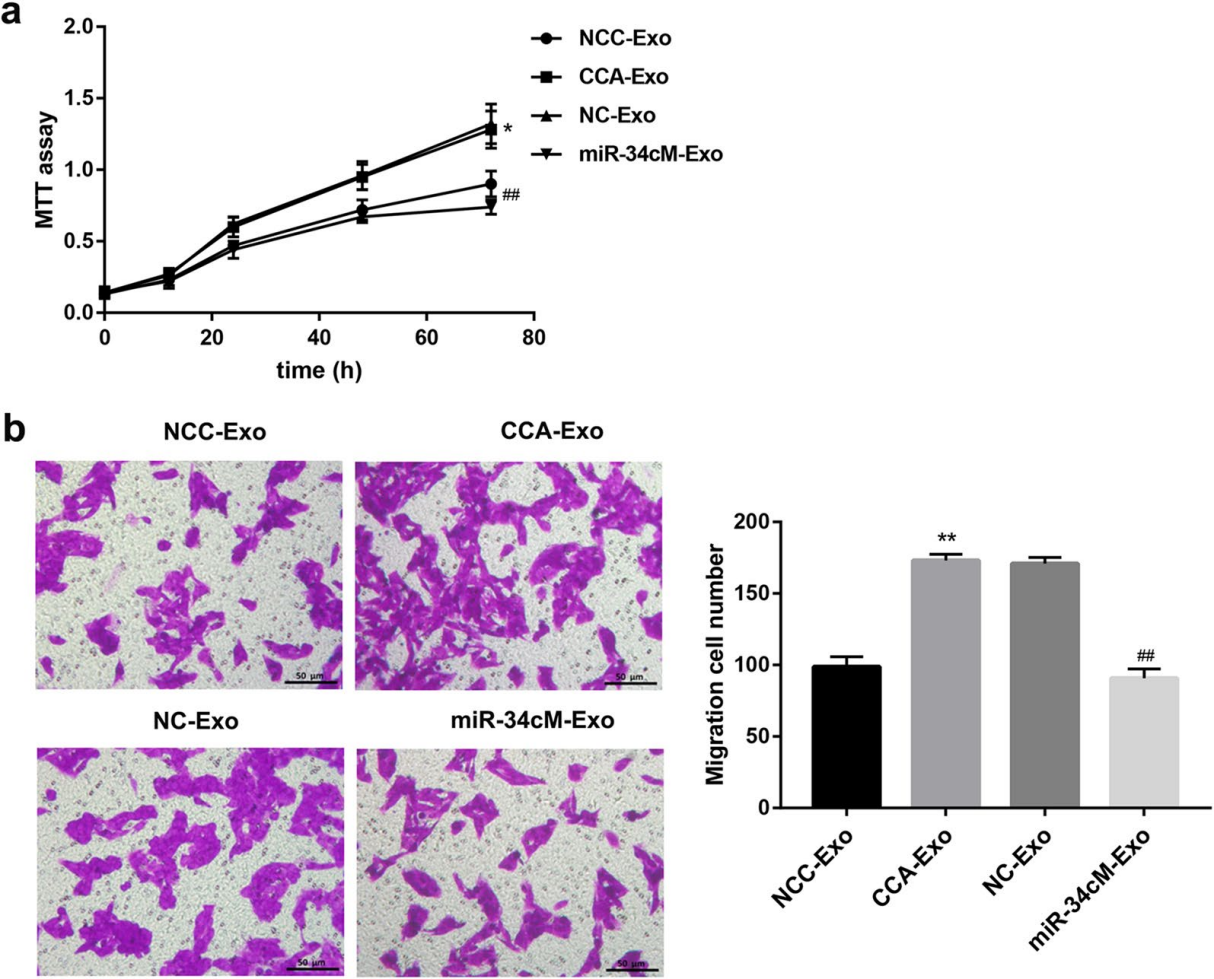
Activated fibroblasts regulate cell proliferation and migration in QBC939 cells via exosomal miR-34c. **a**, **b** MTT and migration assays analysis of cell proliferation and migration in QBC939 cells treated with exosomes from HIBEC and HuCCT-1 cells transfected with or without miR-34c mimics or control; Scale bar, 50 µm. *P < 0.05, **P < 0.01 *vs.* inhibitor NCC-Exo groups; ^##^P < 0.01 *vs.* NC-Exo groups

**Fig. 7:**
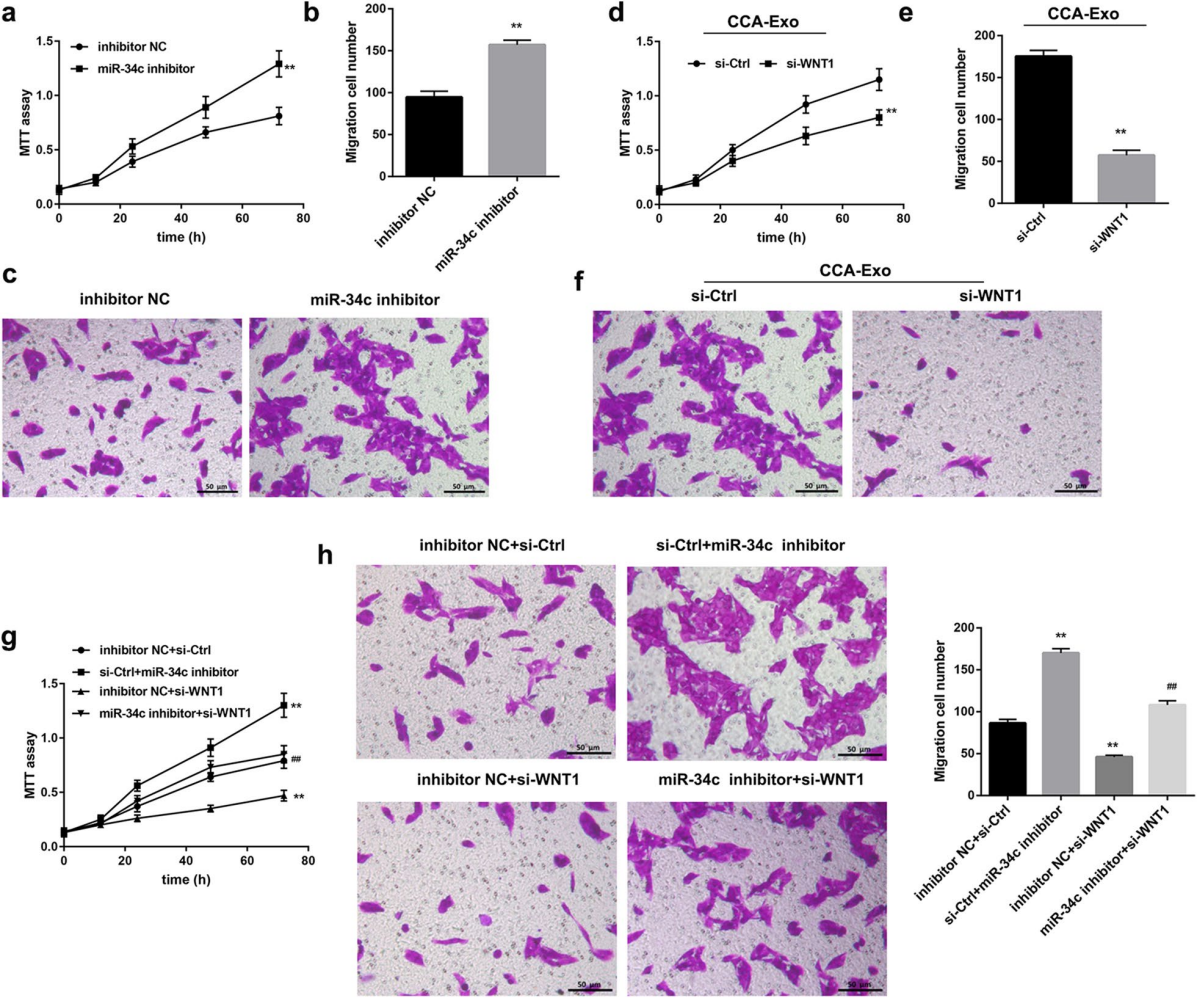
Activated fibroblast affects cell proliferation and migration via miR-34c–WNT1 axis. Cell proliferation and migration have been assessed in multiple cell types in co-cultures—QBC939 cells treated with supernatant of CCC-HSF-1 cells treated with miR-34c inhibitor or si-WNT1. **a-c** MTT and migration assay analysis to assess cell proliferation and migration in QBC939 cells treated with or without miR-34c inhibitor. **d-f** MTT and migration assay analysis to detect cell proliferation and migration in QBC939 cells treated with or without si-WNT1 + CCA-Exo. **g**, **h** MTT and migration assay analysis to determine cell proliferation and migration in QBC939 cells treated with miR-34c inhibitor and/or WNT1 siRNA; Scale bar, 50 µm. *P < 0.05, **P < 0.01 *vs.* inhibitor NC + si-Ctrl groups; ^##^P < 0.01 *vs.* si-Ctrl + miR-34c inhibitor NC groups or inhibitor NC+si-WNT1

**Fig. 8:**
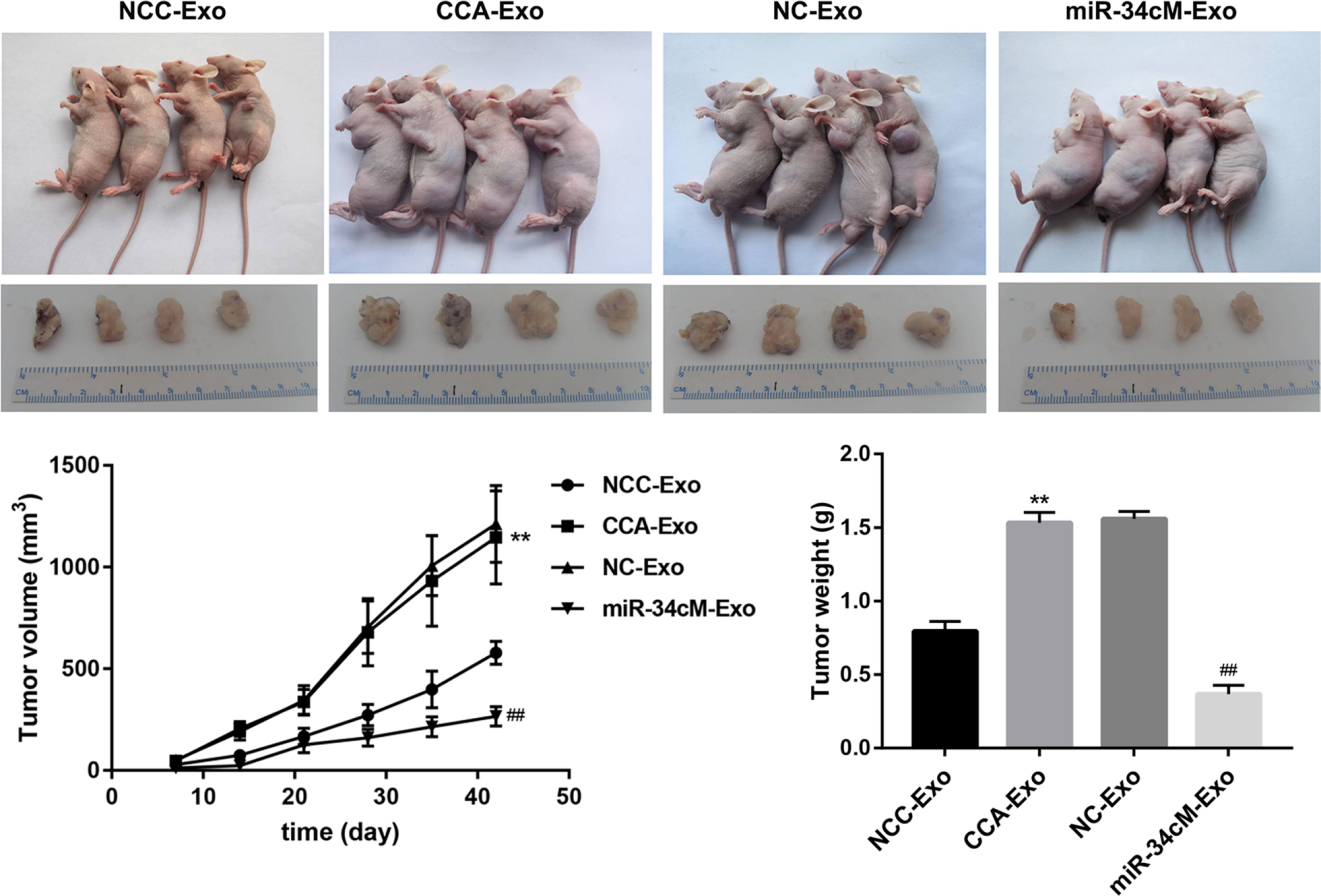
Activated fibroblasts promote cholangiocarcinoma progression via miR-34c. Xenograft tumorigenicity assay for nude mice injected with QBC939 cells pretreated with exosomes from CCC-HSF-1 cells incubated with or without miR-34c mimics. Representative tumors (upper panel) and measurement of tumor volume and weight (lower panel) are shown. **P < 0.01 *vs.* NCC-Exo; ^##^P < 0.01 *vs.* NC-Exo groups

Firstly, we detected expression of miR-34c and Wnt1 in CCC-HSF-1 cells, CCA-Exo and co-culture of CCC-HSF-1 cells and CCA-Exo (Additional file 5). Further, we performed in vitro and in vivo experiments to determine whether fibroblasts activated by suppression of miR-34c contribute to tumor development. First, CCC-HSF-1 cells were incubated with exosomes from NCC- and CCA-group for 24 h, after which the supernatant was used to treat the QBC939 cells. We found that exosomes derived from cancer cells stimulated cell proliferation and migration in QBC939 cells, whereas, over-expression of miR-34c suppressed the effect of these exosomes on cell proliferation and migration than in control group (Fig. 6a, b). Furthermore, QBC939 cells were incubated with the supernatant of fibroblasts pretreated with miR-34c inhibitor or si-WNT1 and CCA-Exo. The results showed that the ability of cell proliferation and migration of QBC939 cells was increased in miR-34c inhibitor group, but decreased in si-WNT1 group, than that in control groups, respectively (Fig. 7a–f). Moreover, WNT1 silencing reversed the effect of inhibition of miR-34c in QBC939 cells (Fig. 7g, h). Additionally, similar results were obtained in the tumorigenicity assay with nude mice (Fig. 8), indicating that exosomes deliver miR-34c to the cancer cells to repress tumor growth. Taken together, these results indicate that exosomal miR-34c is delivered via the gap junctional intercellular communication between the tumor cells and fibroblasts to restrain progression in cholangiocarcinoma.

## Discussion

Complex networks of interacting intercellular and intracellular signals play a critical role in the occurrence and development of cancers [36, 37]. Moreover, the interactions between cancer cells and their microenvironment impact tumor growth, dormancy, invasion, and metastasis [38–40]. It is also widely accepted that cancer-derived exosomes influence the local stroma, and drive the production of a disease-associated microenvironment [41, 42]. Changes in the TME are related to the high and low malignancy of tumor by triggering the activation of otherwise cells in microenvironment to facilitate to cancer cells malignant process. Recent studies have suggested that cancer-derived exosomes trigger differentiation of stromal cells into CAFs [43]. CAF has been reported to have a high expression of ASMA and the level of ASMA in CCA tissues [44]. Cancer-related inflammation plays a prominent role in the effect of TME on pro-tumorigenic action [45]. Here, we found that the pro-inflammatory factors—IL-1β, IL-6, and IL-8—were significantly increased in fibroblasts treated with exosomes derived from tumor cells. Moreover, levels of CAF marker proteins and cell migration were increased in the exosome-treated group than that in control group. Thus, exosomes derived from HuCCT-1 cells could induce activation of CAFs.

Several previous studies have showed the paracrine function of CAFs in activating CCA cells and controlling cancer growth, invasion and resistance [46–48]. Furthermore, majority of the studies suggest miRNAs to be directionally regulated in CAFs, and involved in tumorigenesis and malignant progression [49–51]. The exosome-containing miRNAs contribute to cancer development, including cancer initiation, proliferation, invasion, metastasis, and induction of angiogenesis [29, 30, 52, 53]. In the present study, the CCC-HSF-1 cells treated with HuCCT-1–derived exosomes, containing elevated levels of miR-34c, showed significant decrease in expression of IL-1β, IL-6, IL-8, α-SMA, and FAP. Moreover, overexpression of miR-34c suppressed the migratory potential of fibroblasts. These results suggest that miR-34c fibroblasts are activated by exosomal-based delivery system in cell-to-cell signaling.

Next, we identified WNT1 as one of the target genes of miR-34c, and also confirmed the binding site. The WNT1 signaling pathway is known to play an important role in regulating the activation of cancer-associated fibroblasts [54, 55], and this may explain the regulatory effect of miR-34c on activation of fibroblasts. However, the exact mechanism by which miR-34c–WNT1 activates CAFs remained to be elucidated, and further research will be required. Further, inhibition of miR-34c significantly elevated the expression of pro-inflammatory genes and fibroblast-related proteins, and promoted cell migration; but, increased expression of WNT1 reversed the effect of miR-34c. Thus, these findings suggest that the miR-34c–WNT1 axis in CCFs may influence the progression of CCA cells. Additionally, exosomes show attributes of intercellular communication in regulating the functions of recipient cells via paracrine and endocrine mechanisms, which transport bioactive molecules [56]. Consistent with this, we found that inhibition of miR-34c in fibroblasts promoted the cell proliferation and migration in QBC939 cells, although WNT1 suppression restrained the effect of miR-34c on tumor progression, in vitro and in vivo.

## Conclusions

In conclusion, we found that exosomes exhibit increased secretion of IL-1β, IL-6, and IL-8 in CAFs, promoting fibroblast activation, stimulation of Wnt signaling pathway, tumor cell proliferation and migration, and cancer growth. Moreover, high expression of miR-34c suppressed tumor growth. Thus, the data suggest that downregulation of tumor-derived exosomal miR-34c can transform fibroblasts into CAFs via targeted modulation of WNT1 to activate the Wnt signaling pathway in CCA. These results may aid in elucidating the underlying mechanism of communication between tumor cells and fibroblasts to promote progression in CCA. Additionally, the study findings would allow generation of efficient preventive and therapeutic strategies in CCA.

## Supplementary information

**Additional file 1.** Isolation and identification of the exosomes. Representative TEM images for exosomes derived from HuCCT-1 and HIBEC cells; Scale bar, 500 nm.

**Additional file 2.** Identification of th exosomes (a) Nanoparticle tracking assay-based analysis of exosome size distribution. (b) Western blotting analysis of exosomal markers CD9, CD63, and CD81.

**Additional file 3** ELISA analysis of expression of IL-1, IL-6 and IL-8 in CCC-HSF-1 cells supernatant. (a) ELISA analysis of IL-1, IL-6 and IL-8 in CCC-HSF-1 cells treated with exosomes from HuCCT-1 or HIBEC cells. *P < 0.05 vs. NCC-Exo (b) ELISA analysis of IL-1, IL-6, and IL-8 in exosomes derived from HuCCT-1 cells treated with or without miR-34c mimics.*P < 0.05 vs. NC-Exo groups. (c) ELISA analysis of IL-1, IL-6 and IL-8 in CCC-HSF-1 cells treated with or without miR-34c inhibitor. *P < 0.05 vs. inhibitor NC. (d) ELISA analysis of IL-1, IL-6 and IL-8 in CCC-HSF-1 cells transfected with miR-34c inhibitor and/or Wnt1 siRNA. *P < 0.05, vs. inhibitor NC + si-Ctrl groups or inhibitor NC + si-Ctrl groups; #*P* < 0.05 vs. si-Ctrl + miR-34c inhibitor NC groups orinhibitor NC+si-WNT1.

**Additional file 4.** Identification of siRNA to WNT1 gene. (a) qRT-PCR analysis of expression of WNT1 in all si-WNT1. (b) Migration assays of CCC-HSF-1 cells transfected with WNT1-1 or control.**P < 0.01 vs. si-Ctrl.

**Additional file 5.** The basal level of miR-34c and WNT1 in CCC-HSF-1 cells with or without treatment of exosome. (a) qRT-PCR analysis of expression of miR-34c and WNT1. (b) Western blot analysis of WNT1.**P < 0.01 vs. CCC-HSF-1 group.

## Data Availability

The datasets used and/or analysed during the current study are available from the corresponding author on reasonable request.
