# Supplementary figures and images for: Downregulation of tumor‐derived exosomal miR-34c induces cancer‐associated fibroblast activation to promote cholangiocarcinoma progress
Source: Cancer Cell Int. 2021 Jul 14;21:373. doi: 10.1186/s12935-020-01726-6 (PMC8278610; doi:10.1186/s12935-020-01726-6)

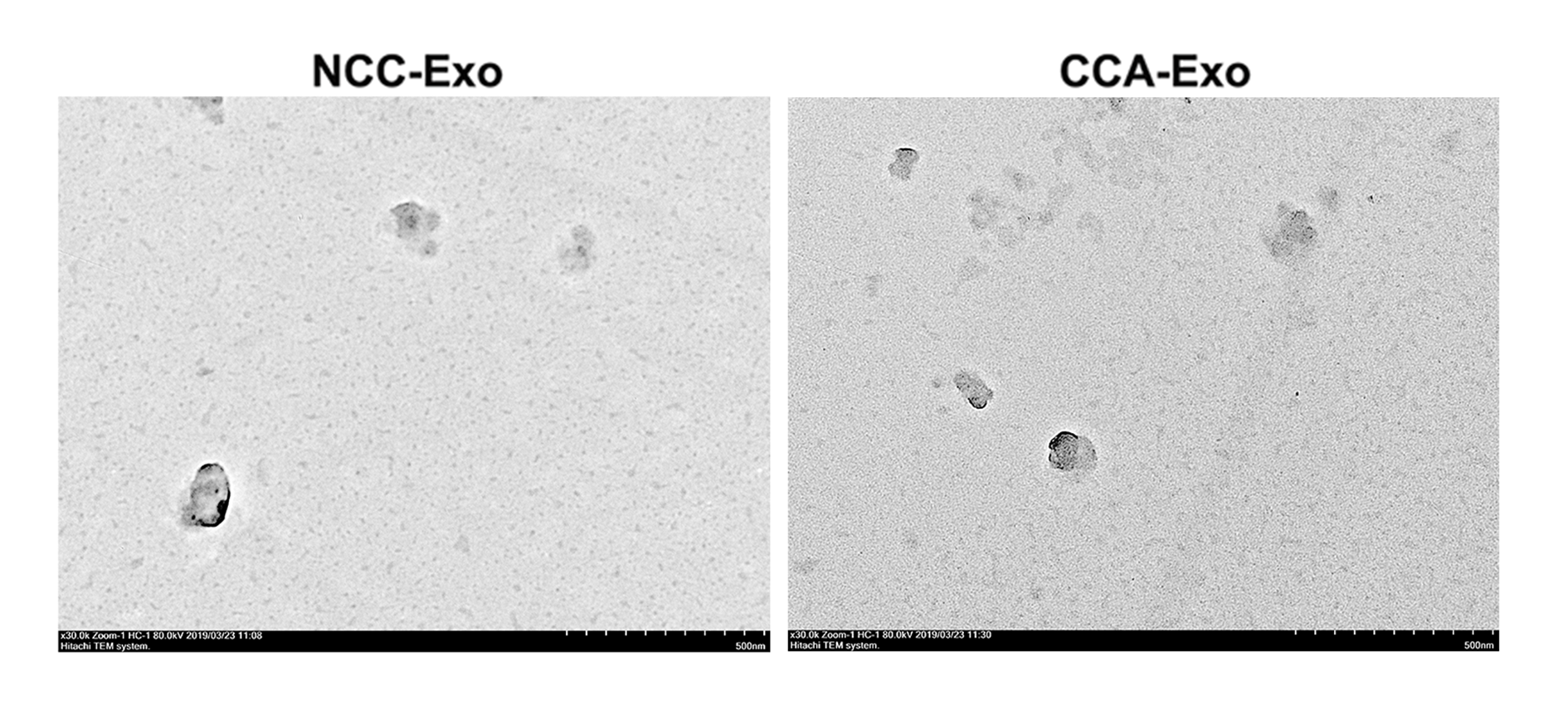

Supplement: Supplementary file 1 — Additional file 1. Isolation and identification of the exosomes. Representative TEM images for exosomes derived from HuCCT-1 and HIBEC cells; Scale bar, 500 nm. [file 12935_2020_1726_MOESM1_ESM.tif]

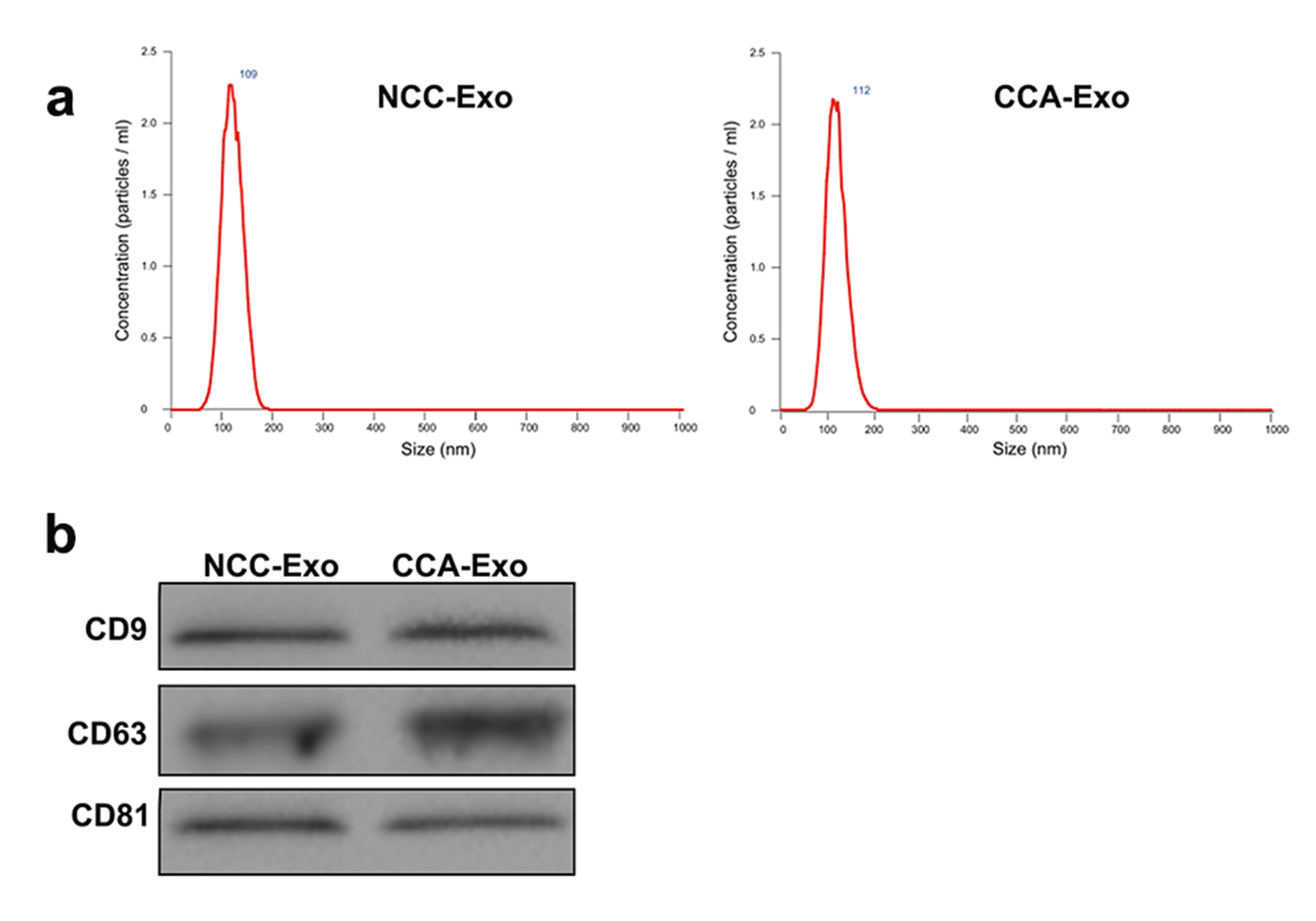

Supplement: Supplementary file 2 — Additional file 2. Identification of th exosomes (a) Nanoparticle tracking assay-based analysis of exosome size distribution. (b) Western blotting analysis of exosomal markers CD9, CD63, and CD81. [file 12935_2020_1726_MOESM2_ESM.tif]

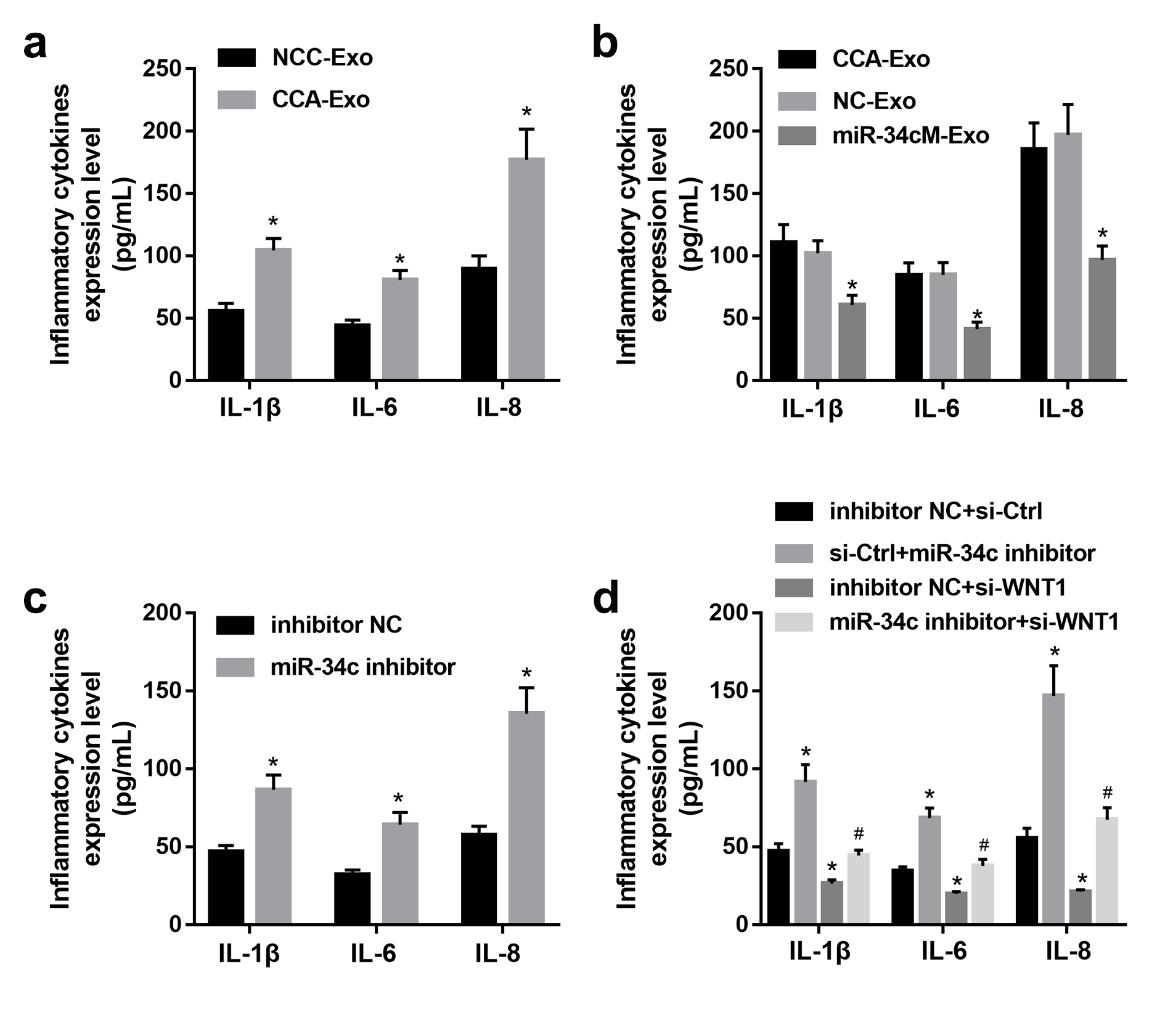

Supplement: Supplementary file 3 — Additional file 3 ELISA analysis of expression of IL-1, IL-6 and IL-8 in CCC-HSF-1 cells supernatant. (a) ELISA analysis of IL-1, IL-6 and IL-8 in CCC-HSF-1 cells treated with exosomes from HuCCT-1 or HIBEC cells. *P < 0.05 vs. NCC-Exo (b) ELISA analysis of IL-1, IL-6, and IL-8 in exosomes derived from HuCCT-1 cells treated with or without miR-34c mimics.*P < 0.05 vs. NC-Exo groups. (c) ELISA analysis of IL-1, IL-6 and IL-8 in CCC-HSF-1 cells treated with or without miR-34c inhibitor. *P < 0.05 vs. inhibitor NC. (d) ELISA analysis of IL-1, IL-6 and IL-8 in CCC-HSF-1 cells transfected with miR-34c inhibitor and/or Wnt1 siRNA. *P < 0.05, vs. inhibitor NC + si-Ctrl groups or inhibitor NC + si-Ctrl groups; #P < 0.05 vs. si-Ctrl + miR-34c inhibitor NC groups orinhibitor NC+si-WNT1. [file 12935_2020_1726_MOESM3_ESM.tif]

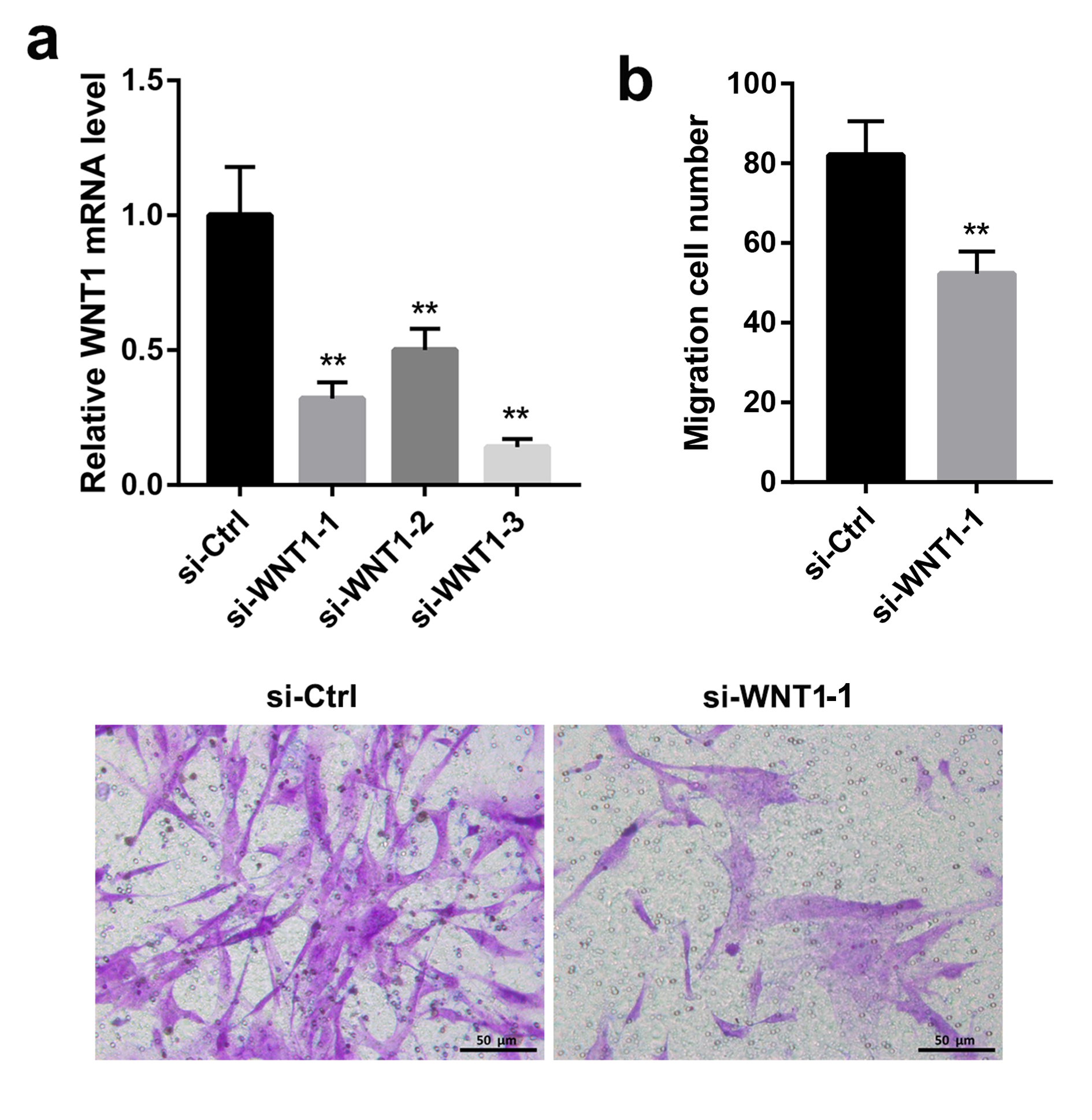

Supplement: Supplementary file 4 — Additional file 4. Identification of siRNA to WNT1 gene. (a) qRT-PCR analysis of expression of WNT1 in all si-WNT1. (b) Migration assays of CCC-HSF-1 cells transfected with WNT1-1 or control.**P < 0.01 vs. si-Ctrl. [file 12935_2020_1726_MOESM4_ESM.tif]

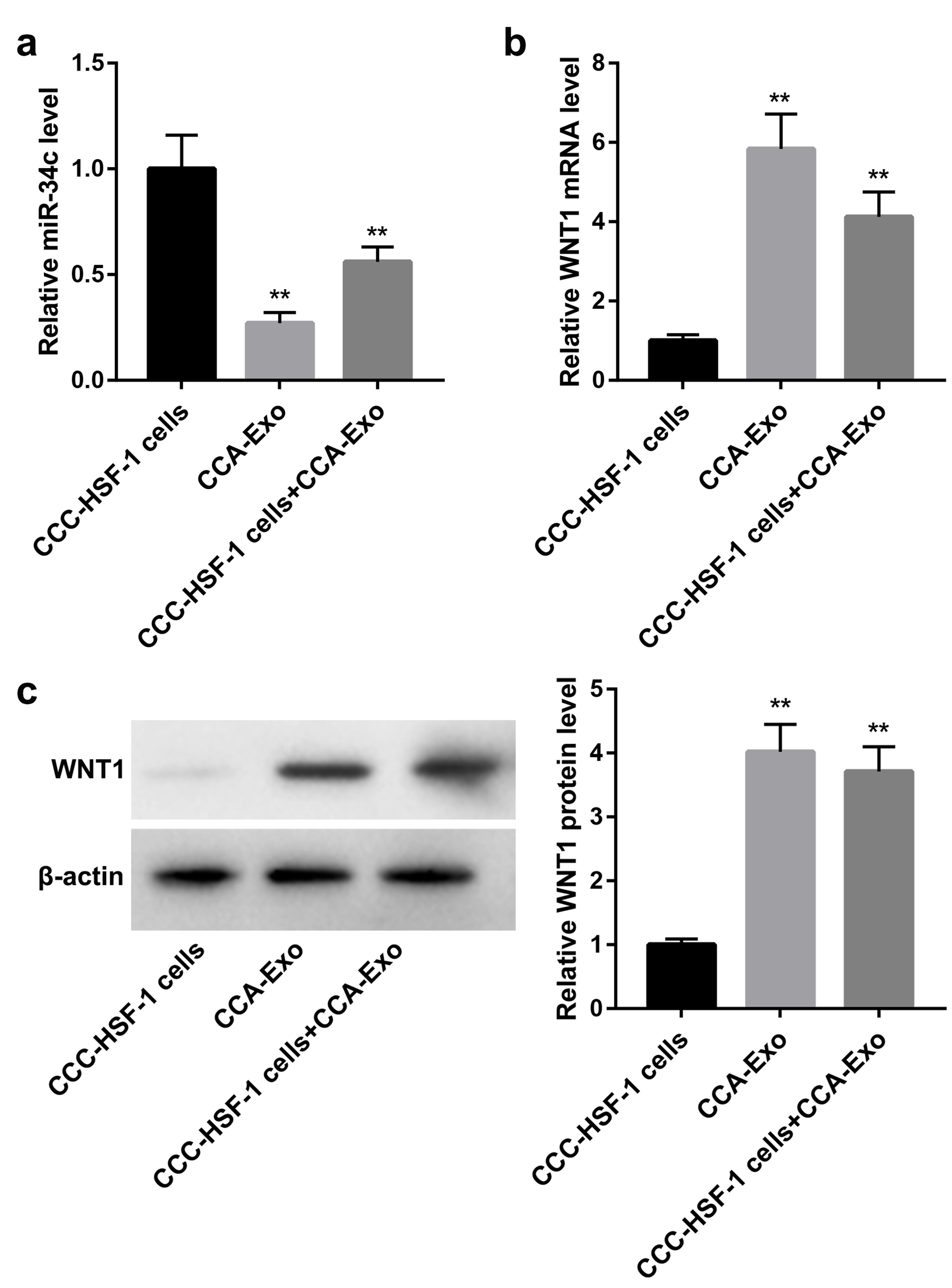

Supplement: Supplementary file 5 — Additional file 5. The basal level of miR-34c and WNT1 in CCC-HSF-1 cells with or without treatment of exosome. (a) qRT-PCR analysis of expression of miR-34c and WNT1. (b) Western blot analysis of WNT1.**P < 0.01 vs. CCC-HSF-1 group. [file 12935_2020_1726_MOESM5_ESM.tif]
